# The Current Status of the Ketogenic Diet in Psychiatry

**DOI:** 10.3389/fpsyt.2017.00043

**Published:** 2017-03-20

**Authors:** Emmanuelle C. S. Bostock, Kenneth C. Kirkby, Bruce V. M. Taylor

**Affiliations:** ^1^Psychology, School of Medicine, University of Tasmania, Hobart, TAS, Australia; ^2^Psychiatry, School of Medicine, University of Tasmania, Hobart, TAS, Australia; ^3^Menzies Institute for Medical Research, Tasmania, Hobart, TAS, Australia

**Keywords:** ketogenic diet, psychiatry, mental disorders, ketones, epilepsy

## Abstract

**Background:**

The ketogenic diet (KD) has been used in treatment-resistant epilepsy since the 1920s. It has been researched in a variety of neurological conditions in both animal models and human trials. The aim of this review is to clarify the potential role of KD in psychiatry.

**Methods:**

Narrative review of electronic databases PubMED, PsychINFO, and Scopus.

**Results:**

The search yielded 15 studies that related the use of KD in mental disorders including anxiety, depression, bipolar disorder, schizophrenia, autism spectrum disorder (ASD), and attention deficit hyperactivity disorder (ADHD). These studies comprised nine animal models, four case studies, and two open-label studies in humans. In anxiety, exogenous ketone supplementation reduced anxiety-related behaviors in a rat model. In depression, KD significantly reduced depression-like behaviors in rat and mice models in two controlled studies. In bipolar disorder, one case study reported a reduction in symptomatology, while a second case study reported no improvement. In schizophrenia, an open-label study in female patients (*n* = 10) reported reduced symptoms after 2 weeks of KD, a single case study reported no improvement. In a brief report, 3 weeks of KD in a mouse model normalized pathological behaviors. In ASD, an open-label study in children (*n* = 30) reported no significant improvement; one case study reported a pronounced and sustained response to KD. In ASD, in four controlled animal studies, KD significantly reduced ASD-related behaviors in mice and rats. In ADHD, in one controlled trial of KD in dogs with comorbid epilepsy, both conditions significantly improved.

**Conclusion:**

Despite its long history in neurology, the role of KD in mental disorders is unclear. Half of the published studies are based on animal models of mental disorders with limited generalizability to the analog conditions in humans. The review lists some major limitations including the lack of measuring ketone levels in four studies and the issue of compliance to the rigid diet in humans. Currently, there is insufficient evidence for the use of KD in mental disorders, and it is not a recommended treatment option. Future research should include long-term, prospective, randomized, placebo-controlled crossover dietary trials to examine the effect of KD in various mental disorders.

## Introduction

The ketogenic diet (KD) has a long-standing place in neurology and has been used for treatment-resistant epilepsy since the 1920s (1). KD consists of a rigidly controlled high-fat, low-protein, and low-carbohydrate diet usually with a 4:1 lipid:non-lipid ratio (fat to protein and carbohydrate ratio) (2). Woodyatt noted that in a normal person in a state of starvation or eating a diet containing low carbohydrate and a high percentage of fat, the ketones acetone, acetoacetate, and beta-hydroxybutyric acid increase (3), and the absence of glucose serves as alternative fuels for the body. KD has been proven an effective treatment in difficult-to-control seizures with its use primarily in children with epilepsy (4, 5), particularly those with epileptic encephalopathies whereby epileptic activity may contribute to severe neurological and cognitive impairments (6). The finding that KD is beneficial for epilepsy was supported by a systematic review (7), meta-analysis (8), and a Cochrane review (9). KD and related diets have been proven useful in pharmacoresistant childhood epilepsy (10).

The mechanism by which KD acts is not clearly understood. However, among the many hypotheses advanced, elevation of brain acetone may account for the efficacy of the diet in epilepsy as it has proven anticonvulsant effects (11). In a variation of the diet, the medium-chain triglyceride (MCT) KD increases plasma levels of decanoic acid, which *in vivo* has been shown to be anticonvulsant; although the precise mechanism remains unclear (12). In young and adult rats, KD increases concentrations of kynurenic acid (KYNA) in the hippocampus and striatum but not the cortex (13). Elevated levels of KYNA in the cerebrospinal fluid have been demonstrated in patients with schizophrenia (14) and bipolar disorder (15). Pharmacological manipulation of kynurenines is a potential treatment strategy for psychiatric disorders (16).

Currently, there are no international protocols guiding the implementation of the diet, rather dietary recommendations are based on individual treating physician’s advice. Consequently, there exists a need for more standardized protocols for management recommendations for clinical and research use (17). In 2006, a group of 26 pediatric epileptologists and dieticians was convened to create a consensus statement regarding the clinical management of KD. They specified the following absolute contraindications to commencing KD “carnitine deficiency (primary), carnitine palmitoyltransferase (CPT) I or II deficiency, carnitine translocase deficiency, beta-oxidation deficiencies including medium-chain acyl dehydrogenase deficiency (MCAD), long-chain acyl dehydrogenase deficiency (LCAD), short-chain acyl dehydrogenase deficiency (SCAD), long-chain 3-hydroxyacl-CoA deficiency, medium-chain 3-hydroxyacl-CoA deficiency, pyruvate carboxylase deficiency and porphyria. Relative contraindications of KD include the following: inability to maintain adequate nutrition, surgical focus identified by neuroimaging and video EEG monitoring, and parent or caregiver non-compliance” (18). The possible risks of KD must be weighted against its potential value for seizure control or its other benefits (19).

Ketogenic diet has been assessed in a variety of neurological conditions other than epilepsy in both animal models and human trials. In an animal model of amyotrophic lateral sclerosis, SOD1-G93A transgenic mice were fed KD. It was shown that KD led to significant alterations in the clinical manifestation of the disease, specifically a higher motor neuron count in the lumbar spinal cord and preserved motor function (20). KD has also been trialed in rats following controlled cortical impact injury, a model for brain trauma, showing that the diet improves both cognitive and motor functioning (21). In an animal model of multiple sclerosis, the effects of KD on memory impairments and inflammation expressed by experimental autoimmune encephalomyelitis were examined. In mice, it was demonstrated that brain inflammation was associated with impaired spatial learning and memory function, and the administration of KD exerted protective effects against these. The proposed mode of action was through attenuation of the immune response and increased oxidative stress observed in the mice (22).

In humans, KD has been trialed in a number of neurological conditions. In a randomized, double-blind, placebo-controlled, parallel group study in Alzheimer’s disease, an oral ketogenic compound AC-1202 was tested on 152 patients. Regular medications were continued throughout the study. Daily dosing of AC-1202 significantly elevated the levels of beta-hydroxybutyrate 2 h after administration. After 45 and 90 days, patients treated with AC-1202 had significant improvements on the ADAS-Cog scale (23). In a small study of seven patients with Parkinson’s disease, five adhered to KD for 28 days (24). Scores on the Unified Parkinson’s Disease Rating Scale improved in all five as did symptoms such as resting tremor, freezing, balance, gait, mood, and energy levels. These results should be interpreted with caution due to the small sample size, subjective ratings, and the lack of a control group to exclude a placebo effect. The modified Atkins diet (a high-fat, low-carbohydrate diet), which creates a ketotic state was trialed in adolescent patients with chronic daily headaches (25). Due to difficulties adhering to the diet, the study was terminated prematurely. Three participants reported an improvement in headache severity and quality of life; however, they still required pharmacotherapy to manage their condition. In a comprehensive review of KD in diverse neurological conditions, Stafstrom and Rho concluded that there are rich opportunities for further investigation of KD in both the laboratory and clinical practice (26).

The therapeutic advantage of KD has been replicated in animal models of neurological illnesses, and the purported underlying mechanisms include those which improve mitochondrial function (27). Molecular, biochemical, and physiological studies tend to support the assumption that cellular energy status is a determinant for multiple disorders (28). Aberrant energy production has been associated with cancer (29), heart failure (30), aging (31), and neurological conditions such as epilepsy (32) and Alzheimer’s disease (33). The precise pathways by which energy disruption is related to these and other disorders are unknown. There are also strong indications of metabolic pathways involving energy production in the pathophysiology of some mental disorders including bipolar disorder, depression, schizophrenia (34) autism spectrum disorder (ASD) (35), and potentially attention deficit hyperactivity disorder (ADHD) (36). There is also a recognized comorbidity between epilepsy and mental disorders (37), which might indicate some commonality of mechanisms.

Given the degree of interest in KD and neurological conditions, the aim of this narrative review is to examine the effect of the diet in mental disorders. The literature searched in anxiety, depression, bipolar disorder, schizophrenia, ASD, and ADHD.

## Method

A comprehensive search of the electronic databases PubMed, PsychINFO, and Scopus for peer-reviewed articles published in English was conducted in the last week of November 2016 and updated in January 2017. Search terms were “bipolar disorder” “manic depress*” “depress*” “schizophren*” “autism” “ASD” “attention deficit hyperactivity disorder” “ADHD” “obsessive compulsive disorder” “OCD” “anxiety” “anxi*” “psychiatry” “mental disorder*” (group 1) AND “ketogenic diet” “ketosis” “ketogenesis” “ketone bodies” “high fat low carbohydrate” “diet” “acetone” “acetoacetic acid” “beta-hydroxybutyric acid” “acetyl-coA” “ketonemia” “ketonuria” “fatty acid metabolism” “hyperketonemia” “fasting” “nutritional ketosis” “acidotic” (group 2). These terms were combined as follows: group 1 AND group 2. In addition, a hand-search of the reference lists of published articles was also conducted, and articles were assessed for their suitability in the review. An initial search was conducted using all the search terms listed above, and abstracts were reviewed by author Emmanuelle C. S. Bostock. Full text publications were retrieved for those that addressed the subject matter.

## Results

The results are discussed by mental disorders examining animal and human studies including case reports and studies of patient groups. The search yielded 15 studies that examined KD in mental disorders, specifically anxiety, depression, bipolar disorder, schizophrenia, autism, and, ADHD. These studies included nine animal models, and in humans four case studies and two uncontrolled trials. A summary of results by animal models and human studies are presented in Tables 1 and 2, respectively.

**Table 1:** **Summary of findings in animal models**.

Reference	Condition	Subjects (*n*)	Mode of administration of diet	Duration of diet	Ketone*	Result
(38)	ANX	Sprague-Dawley (48) and Wistar Albino Glaxo/Rijswijk rats (32)	Exogenous ketone supplement	83 or 7 days *via* oral gavage	✓	Reduced ANX-related behavior

(39)	DEP	Wistar rats (20)	4:1 lipid:non-lipid ratio	7 days	✓	Some evidence for potential antidepressant properties

(40)	DEP	CD-1 mice (20)	4:1 lipid:non-lipid ratio *in utero* and SD in postnatal life	30 days	✓	Those fed KD *in utero* showed reduced susceptibility to ANX and depression and increased hyperactivity

(41)	SZ	C57Bl/6 mice (?)	77.6% fat, 9.5% protein, and 4.7% crude fiber, AD fiber 4.7%	3 weeks	✓	Normalized pathological behaviors including psychomotor hyperactivity, stereotyped behavior, social withdrawal, and working memory deficits

(42)	ASD	Swiss mice (16)	(Lard 690 g/kg, sunflower oil 5 g/kg, protein 250 g/kg, fiber 10 g/kg, ash 5 g/kg)	*In utero* exposure to KD (70 days)	–	Statistically significant social deficits and stereotypies that are common behaviors in those with ASD

(43)	ASD	Wistar rats (6)	6:1 lipid:non-lipid ratio	10–14 days	✓	KD had a significant effect and was able to modify complex social behaviors in valproic acid and control rats

(44)	ASD	BTBR mice (?)	6.3:1 lipid:non-lipid ratio	14 days	✓	Temporal cortex and hippocampus brain regions showed improvements on autistic deficits associated with myelin formation and white matter development

(45)	ASD	EL mice (?)	3.0:1 or 6.6:1 lipid:non-lipid ratio	3–4 weeks	✓	Social novelty test—females fed higher KD ratio exhibited significant preference to the new mouse. Self-grooming significantly decreased in males

(36)	ADHD	Dogs (21)	10% moisture, 28% protein, 15% fat, 6% ash, 2% crude fiber, and MCT oil	6 months	✓	Significant improvement in ADHD-related behaviors

ANX, anxiety; DEP, depression; BD, bipolar disorder; SZ, schizophrenia; *, ketone levels reported; ?, unknown sample size; MCT, medium-chain triglyceride; ASD, autism spectrum disorder; ADHD, attention deficit hyperactivity disorder; KD, ketogenic diet.

**Table 2:** **Summary of findings in human studies**.

Reference	Condition	Subjects (*n*)	Mode of administration of diet	Duration of diet	Ketone*	Result
(46)	BD	Human women (2)	Ratio not mentioned in first but in second (70% fat, 22% protein, and 8% carbohydrate)	2 and 3 years	✓	Mood stabilization

(47)	BD	Human woman (1)	4:1 lipid:non-lipid ratio	1 month	No urinary ketones detected	No clinical improvement

(48)	SZ	Human women (10)	Not listed	2 weeks	Not listed	Statistically significant decrease in symptomatology

(49)	SZ	Human woman (1)	Not listed	12 months	Not listed	No recurrence of auditory or visual hallucinations

(50)	ASD	Human children (30)	30% MCT, 30% fresh cream, 11% saturated fat, 19% carbohydrate, and 10% protein	6 months (intervals of 4 weeks with 2 diet-free weeks)	✓	40% non-compliance. Two children showed significant improvements on Childhood Autism Rating Scale, while the rest showed mild-to-moderate improvements

(51)	ASD	Human child (1)	1.5:1 lipid:non-lipid ratio	Several years	✓	Score on the Childhood Autism Rating Scale decreased from 49 to 17 (severe autism to non-autistic)

DEP, depression; BD, bipolar disorder; SZ, schizophrenia; *, ketone levels reported; MCT, medium-chain triglyceride; ASD, autism spectrum disorder.

### Anxiety

Anxiety is a common mental disorder affecting 18.1% of the population in the United States (52). In humans, functional magnetic resonance imaging indicates that anxiety is associated with activation in the ventromedial prefrontal cortex and hippocampal regions of the brain (53). Symptoms of anxiety and disorders are more frequent in patients with epilepsy with one recent study reporting a lifetime incidence of 22.8% as opposed to 11.2% in people without epilepsy (54).

In a recent animal model study of anxiety in male rats, two methods of administration of exogenous ketone supplement were applied (38). In the chronic administration condition, 48 male Sprague-Dawley (SPD) rats were fed for 83 days with either a standard diet (*n* = 9) or standard diet plus one of four ketone supplementation conditions. In the sub-chronic intragastric gavage bolus condition, 39 SPD rats were fed with standard diet and gavaged daily with water (control, *n* = 11) or 1 of 3 levels of ketone supplementation for 7 days; this was repeated with 32 Wistar Albino Glaxo/Rijswijk rats receiving a half-dose of supplementation. In both modes of supplementation, beta-hydroxybutyrate was significantly elevated indicating ketosis. All treatment conditions resulted in reduced anxiety as assessed by behavior on the elevated plus maze. The dependent variables of less entries and time spent in closed arms, more entries and time spent in open arms, more distance traveled in open arms, and delayed entry to closed arm were used as an analog of anxiety in humans. The authors hypothesized that the mode of action was through the glutamatergic and/or GABAergic and purinergic systems.

### Depression

In a recent review, a number of studies suggested that depression is associated with an increased risk of epilepsy (55). The effectiveness of conventional antidepressant therapies is frequently examined in animals. In rodents, to test current levels of depression, a methodology known as the Porsolt forced swim test is often employed (56) and has been used in testing the effectiveness of new antidepressant drugs (57). In the two-part swim test, animals are first placed in a container from which they cannot escape. When they then stop trying and immobility ensues, a state of behavioral despair is shown. Second, to assess the effects of antidepressants, the time spent immobile is used as a dependent variable, and reductions are interpreted for significance (56). To examine the antidepressant properties of KD, 20 Wistar rats given the diet (4:1 lipid:non-lipid ratio) were compared to 20 fed a standard diet (39). It was found that rats on KD spent less time immobile than control rats thus providing some evidence for potential antidepressant effects of the diet. The diet duration was 7 days, and levels of beta-hydroxybutyrate were measured.

Brain morphology and behavior of CD-1 mice exposed to KD (4:1 lipid:non-lipid ratio) for 30 days *in utero* and fed a standard diet in postnatal life were examined (40). Adult mice that were fed the diet *in utero* showed reduced susceptibility to anxiety and depression and exhibited elevated physical activity when compared with control mice fed a standard diet *in utero*. Morphological differences included cerebellar volumetric enlargement by 4.8%, a hypothalamic reduction by 1.39%, and a corpus callosum reduction by 4.77%, as computed relative to total brain volume.

While animal models pave the way for future research in humans, the conclusions that may be made are limited. The mechanism by which KD acts in animal models of depression is unknown; however, in children with epilepsy, KD resulted in significant alterations in levels of serotonin and dopamine neurotransmitters (58), both of which are implicated in anxiety and depression. To the best of our knowledge, there are no studies examining the effects of KD in depressed humans.

### Bipolar Disorder

A diagnosis of bipolar disorder type I requires an episode of mania, which consists of “a distinct period of abnormally and persistently elevated, expansive or irritable mood, lasting at least 1 week (or any duration if hospitalization is necessary)” (59). A diagnosis of bipolar disorder type II requires at least one episode of hypomania. In a study of nutrition and exercise behavior, when compared to patients with schizophrenia or healthy controls, it was found that patients with bipolar disorder were more likely to report risk factors for poor nutrition including difficulty obtaining or cooking food (60). Treatments for bipolar disorder typically include an antipsychotic and a mood stabilizer, and many patients are treated with adjunct anticonvulsants.

In a case study of two women with bipolar disorder type II, the patients maintained ketosis for an extended period of 2 and 3 years, respectively. The women reported subjective mood stabilization, which exceeded that of medication as well as an overall improvement in their condition that they related to ketosis (measured in the urine). Both women tolerated the diet well with few or no side effects reported (46). The ratio of KD was not mentioned in the first case, but in the second it was estimated to be around 70% fat, 22% protein, and 8% carbohydrates.

In a separate case study, a woman with treatment-resistant bipolar disorder was placed on KD (4:1 lipid:non-lipid ratio) and showed no clinical improvement (47). It should be noted that no urinary ketones were detected, the type of bipolar disorder was not listed (type I or type II), and treatment duration limited to 1 month.

These studies illustrate that careful attention should be paid to the intricacies of the diet (such as measuring ketones and calculating macronutrient ratios) to fully examine its efficacy in bipolar disorder, as well as the need for larger well-designed placebo-controlled studies in this area. The mechanism by which KD may be effective in bipolar disorder is based on the hypothesis that acidosis achieved through ketosis reduces intracellular sodium and calcium, both of which are elevated in the disorder (47). Mood stabilizers reduce intracellular sodium in an activity-dependent manner within the context of KD; this is hypothesized as being achieved through the acidification of the blood (46).

### Schizophrenia

Schizophrenia is associated with high levels of morbidity. The precise pathophysiology of the disorder is unknown, and current pharmacological treatment options are limited (61). Animal models of schizophrenia fit into four induction methods including developmental, drug-induced, lesional, or genetic manipulation (62). In a recent drug-induced (MK-801, dizocilpine) animal model of schizophrenia in C57BL/6 mice, it was demonstrated that 3 weeks of KD (77.6% fat, 9.5% protein, and 4.7% crude fiber, AD fiber 4.7%) normalized pathological behaviors (41). These included psychomotor hyperactivity, stereotyped behavior, social withdrawal, and working memory deficits, which reflect the positive, negative, and cognitive symptoms of the disorder. Weight loss was an observed side effect. Elevated levels of the ketone beta-hydroxybutyrate and decreased glucose levels indicated that metabolic adaptation had occurred.

In a 1965 study, the effect of KD was tested in 10 female patients with schizophrenia. All participants were reported to have a poor prognosis and were not treatment responsive at the time. Concurrent therapies remained throughout the duration of the diet including pharmacotherapy and electroconvulsive therapy. The Beckomberga Rating Scale was administered to patients three times during the diet period (2 days, 2 weeks, and 1 week after discontinuation), there was a statistically significant decrease in symptomatology after 2 weeks of established KD (48). This was, however, a small, poorly controlled study, and in addition, the lipid:non-lipid ratios were not detailed, and it was not stated whether ketone levels were measured throughout the study. A further consideration is that the study was conducted in 1965 before the advent of atypical antipsychotics and their metabolic side effects.

In a case study of a 70-year-old overweight woman with a diagnosis of schizophrenia, KD was initiated by her treating physician (49). The patient remained on KD for 12 months and reportedly had no recurrences of auditory or visual hallucinations, and the patient lost weight. The patient reported eating mainly lean proteins and low-carbohydrate vegetables (the lipid:non-lipid ratio was not listed), ketosis was not confirmed and perhaps not established due to the lack of dietary fats listed; therefore, this case report is of indeterminate value.

Some studies suggest that abnormal glucose and energy metabolism may underlie the pathophysiology of schizophrenia, which may provide some potential pointers into the hypothesized mode of action of KD in the disorder (63, 64). Others have noted that abnormal glucose metabolism may occur secondary to antipsychotic medications alongside significant treatment side effects such as weight gain, hyperglycemia, and diabetes (65). The high metabolic risk associated with schizophrenia is due to genetic and environmental factors (66).

### Autism Spectrum Disorder

Features of patients with ASD include compromised social interaction and communication (67). It is estimated that between 5 and 40% of patients with autism will develop epilepsy (68), and while most patients will respond to pharmacotherapy, in one study, 34% of 170 patients had medically refractory epilepsy (69). The precise pathogenesis of ASD remains unknown, but genetic and environmental factors have been known to contribute to its onset. One such factor is exposure to valproic acid (VPA) *in utero*, which is associated with a 12% incidence of ASD in children (70) and is used as an animal model of induction of ASD (42).

Using the animal model of autism induced by prenatal exposure to VPA in mice, the effects of KD were examined. Pregnant Swiss mice received a single intraperitoneal injection of 600 mg/kg of VPA (*n* = 26) or saline (*n* = 18) on gestational day 11. At day 21, 16 VPA treated and 16 control mice were used. Half of each group was fed KD (lard 690 g/kg, sunflower oil 5 g/kg, protein 250 g/kg, fiber 10 g/kg, ash 5 g/kg), while the other received a standard diet. Ketone levels were not measured. After 70 days on KD, a statistically significant result was found in mice with VPA in behaviors such as social deficits and stereotypies that are common behaviors in those with ASD (42). It is also believed that mitochondrial dysfunction may play a role in the onset of ASD (35). Ahn et al. (43) aimed to determine if KD could reverse the social deficits and mitochondrial dysfunction seen in a prenatal VPA animal model of autism using Wistar rats. On postnatal day 21, rats were placed on either KD (6:1 lipid:non-lipid ratio) or standard diet for 10–14 days. Beta-hydroxybutyrate was measured. KD had a significant effect and was able to modify complex social behaviors in VPA and control rats and mitochondrial respiration (43).

Another animal model of autism using the inbred BTBR mouse strain that exhibits three core features of autism, including reduced sociability, communication, and increased repetitive behavior, was studied (71). In another study, 33 genes were differentially expressed in the temporal cortex and 48 in the hippocampus suggesting deficits in the stress response and in neuronal signaling and communication in BTBR mice. After 14 days on KD (6.3:1 lipid:non-lipid ratio), both brain regions showed improvements on autistic deficits associated with myelin formation and white matter development (44). One study has found that in BTBR mice, KD reduces total gut microbial and compositional remodeling of the mouse microbiome providing a potential explanation as to its efficacy in this model (72).

In an animal model with behavioral characteristics of ASD and comorbid epilepsy in male and female EL mice, the effect of KD was assessed (45). Testing occurred at 8–9 weeks postpartum following 3–4 weeks of dietary treatment. Animals were fed either a standard diet or one of two KDs (3.0:1 or 6.6:1 lipid:non-lipid ratio). KD raised ketones in all groups, but the higher fat ratio deepened ketosis. Both KDs significantly increased sociability, time spent in the chamber with another mouse, in females and males. Social novelty, preference for a newly introduced mouse was higher in females fed the higher KD ratio. The test of repetitive behavior (self-grooming) was significantly decreased in males but was non-significant in females. This study provides some intriguing results regarding the effects of sex and KD in a mouse model of ASD and idiopathic epilepsy.

The role of KD in ASD has been examined in a pilot study of 30 children (50). The diet (30% of energy as MCT oil, 30% fresh cream, 11% as saturated fat, 19% carbohydrates, and 10% as protein) was administered for 6 months with intervals of 4 weeks with 2 diet-free weeks. Of the total sample, 40% did not comply or did not tolerate the diet. Urinary ketones were measured. In the remaining sample, two children showed significant improvements on the Childhood Autism Rating Scale, while the rest showed mild-to-moderate improvements. As observed in patients with epilepsy, after the termination of KD the benefits persisted, which raise intriguing questions regarding the effects of plasticity.

In a case study of a child with autism and epilepsy, following standard treatment non-response, the individual was placed on KD (1.5:1 lipid:non-lipid ratio) with adjunct anticonvulsant therapy (51). The patient was in ketosis. After initiation of the diet several benefits ensued including the resolution of morbid obesity and the improvement of cognitive and behavioral features of the disorder. After several years on the diet, the patient’s score on the Childhood Autism Rating Scale decreased from 49 to 17, a change from a rating of severe autism to non-autistic, and IQ increased by 70 points. Fourteen months following the initiation of the diet the patient was also seizure free.

The suggested mechanisms of action of KD in ASD include that it may reduce pain sensitivity through the reduction of glucose and may have anti-inflammatory properties as it reduces swelling and plasma extravasation (42). In a systematic review of KD in ASD it was concluded that the limited number of reports of improvements after treatment with the diet is not sufficient to attest to the practicability of KD as a treatment for the disorder (73).

### Attention Deficit Hyperactivity Disorder

Attention deficit hyperactivity disorder is characterized by a lack of behavioral inhibition and by neuropsychological deficits in four areas, including working memory, self-regulation of affect–motivation–arousal, internalization of speech, and behavioral analysis and synthesis (74). ADHD is the most commonly occurring mental disorder in children and adolescents with epilepsy occurring in 16 (29.1%) of 78 patients (75). Children with ADHD have a high frequency of epileptiform discharges as observed by EEG (76). In a prospective study of children with epilepsy (*n* = 34) on KD it was found that after 1 year on the diet there was a statistically significant improvement of attention and social functioning (77).

There is little evidence examining ADHD and KD, but a 6-month prospective, randomized, double-blinded, placebo-controlled, crossover dietary trial compared the effects of KD (10% moisture, 28% protein, 15% fat, 6% ash, 2% crude fiber, and MCT oil) or a standard diet on behavior in 21 dogs with comorbid ADHD and idiopathic epilepsy (36). It was hypothesized that there were three specific behaviors related to ADHD in dogs including excitability, chasing, and trainability. ADHD in dogs is manifested as inattention and excitability/impulsivity, which have been likened to the disorder in humans (78). When compared with the standard diet, KD resulted in a significant improvement in ADHD-related behaviors. Serum beta-hydroxybutyrate was measured. The mechanisms of behavioral improvements during KD remain unknown. The authors postulated that alterations of energy metabolism in the brain may contribute to behavioral changes. Research into humans with ADHD and KD is lacking.

## Discussion

In neurology, KD is an established treatment option for treatment-resistant epilepsy with evidence from a range of studies including controlled trials. By contrast, KD research in humans with mental disorders, though extending over a 50-year period, has received little attention with few studies other than case reports, small sample size open studies, and no controlled trials. Animal studies have been more systematic, investigating mechanisms as well as outcomes on putative disease analogs in rodents and canines, the latter including randomized controlled trials of KD.

With respect to mechanisms, the pathophysiology of the mental disorders covered in this review is not clearly understood, though impaired metabolism due to mitochondrial dysfunction has been identified as an important substrate (34). This is congruent with findings in neurological conditions, Stafstrom and Rho concluding that energy metabolism changes induced by KD in neurological conditions suggest a final common pathway implicating mitochondrial function (26). KD may also influence neuronal plasticity by modifying neural circuits and cellular properties to normalize function (26). Mitochondrial dysfunction may be relevant in some mental disorders including schizophrenia, ASD, and ADHD, whereas the improvements seen in anxiety, depression, and bipolar disorder may be related to alterations of neurotransmitters.

One other possible mediator of the beneficial effects of KD in mental disorders is the effect on sleep. In a study of 18 children with treatment-resistant epilepsy, after 3 months of KD sleep was reported to be enhanced with a pattern of significant reduction in total night sleep, preservation of slow-wave sleep, increased rapid eye movement (REM) sleep, and decrease in sleep stage 2 (79). The mechanisms by which KD affects sleep is unclear (80), and more studies are necessary to confirm reports that certain dietary patterns and foods improve sleep (81).

Sleep problems and mental disorders are codependent conditions that exacerbate each other and lead to impaired quality of life and increased disability (82). Impairments of sleep are a widespread feature of mental disorders. Anxious patients have been found to have significantly less sleep period time, total sleep time, percentage stage REM and percent stage 4 sleep, shorter latency to stage REM, and greater percent stage 1 sleep than healthy controls (83). REM sleep abnormalities including shortening of REM latency, lengthening of the duration of the first REM period, and heightening of REM density are found in patients with depression (84). In patients with inter-episode bipolar disorder, shorter sleep onset latency and increased REM density has been observed (85). A decrease of REM sleep latency in schizophrenia has been described (86). Individuals with ASD have prolonged sleep latency, more frequent nocturnal awakenings, lower sleep efficiency, increased duration of NREM stage 1 sleep, and decreased deeper stages of NREM sleep (87). In ADHD, disturbed sleep architecture has been described including shorter REM latencies, reduced REM sleep, and increased delta sleep percentage (88). It should also be noted that sleep deprivation can precipitate mania in bipolar disorder and seizures in epilepsy (89) and can be used as a treatment for depression (90). The specific effects of KD on these mental disorder-related sleep symptoms has not been studied in detail, but interactions are likely and may be possible mediators of a therapeutic effect.

In epilepsy, KD acts differently to antiepileptic drugs (AED) in seizure prevention. While AED act directly on ion channels and synaptic processes, KD acts through intermediary metabolic pathways (91). Chang et al. showed that an MCT (palm oil and coconut oil) diet, a variation of KD, reduces seizures in children *via* inhibition on AMPA receptors (12, 92, 93). The questions posed by the literature indicate that the mechanism of action is still unknown, and there may be many potential pathways involved. The mechanism of action appears different from AED and therefore probably psychiatric drugs also, which opens potential avenues for treatment in a manner that may supplement conventional pharmacological treatment approaches. The exact mechanism of action of KD is unclear, and for detailed discussion, see Rogawski et al. (91). Thus, present knowledge indicates that KD exerts its effects on seizure control by mechanisms different from conventional AED and therefore, in psychiatry, this may also be the case although as yet unproven.

There are a number of reasons why the effectiveness of KD in mental disorders remains unproven. In addition to the low number of human studies, the quality of the studies has some significant limitations. Sample sizes are small, there is no control for placebo effects, and the establishment of ketosis is generally lacking with no confirmatory measurement of ketones in three human studies. There are also significant limitations associated with the diet itself including the detailed regimen, unpalatable food choices, side effects, and duration of diet required. There are also no enforced standards as to what constitutes KD in humans with variable lipid:non-lipid ratios reported. KD monotherapy is used in animal models of mental disorders but remains unexamined in human studies. Ten adult patients with epilepsy followed KD monotherapy, and it was concluded that it may be feasible, well tolerated, and an effective long-term alternative (94).

To comply with KD, patients who may be acutely unwell are required to measure food portions to ensure that the macronutrient targets associated with the diet are met, and they may find it difficult to adhere to such a demanding diet (47). This is particularly so for patients with mental disorders where symptoms such as impulsivity in mania, apathy, and reduced appetite in depression, food cravings, and binge eating associated with antipsychotic medications may variously interfere with compliance with KD (95). A mitigating factor to the outcomes in children with epilepsy may be that the diet is typically administered in a hospital setting initially and subsequently, by caregivers.

El-Mallakh and Paskitti have outlined the adverse consequences of KD including constipation, menstrual irregularities, elevated serum cholesterol and triglycerides, hypoproteinemia, hemolytic anemia, elevated liver enzymes, and gall stones (96). Kidney stones have been noted to occur in 1 of 20 children on the diet (97). In a period of almost 2 years, prospective monitoring of 52 children with pediatric epilepsy was conducted. Ten percent of children experienced serious adverse events associated with the diet 1 month after initiation (98). This included presacral and periorbital edema, developmental impairment, and unwanted weight loss in an infant, renal tubular acidosis, viral gastroenteritis, abnormal liver function, and thrombocytopenia. It should be noted that all patients were being treated with concomitant VPA. It was reported in a retrospective study of 158 children with intractable epilepsy that, in 80% emesis, food refusal and hypoglycemia occurred (99).

By definition, KD is confirmed by the production of ketones measured in the blood or urine. In the reviewed literature covering KD in mental disorders, four studies did not report ketone levels, which severely limit comparability across studies and the ability to invoke any consistent mechanism. One study compared whether measuring serum beta-hydroxybutyrate or urinary ketones was superior to monitor KD (100). In humans, it was found that beta-hydroxybutyrate correlated more strongly with a reduction in seizures than urinary ketones; therefore, future studies should measure ketones in the blood. Another issue is that the lipid:non-lipid ratios used were different (see Tables 1 and 2). In a study that compared the efficacy and tolerability of the 3:1 versus the 4:1 lipid:non-lipid ratios, the latter was shown to have a higher seizure-free outcome (2).

One issue when interpreting the results is the levels of evidence in the evidence-based hierarchy. Animal models of mental disorders are considered valuable preclinical tools to investigate the neurobiological basis of a disorder (62). While this may be true, they are nonetheless subject to a number of limitations. One such limitation is the issue of validity, and their use is based on the assumption that humans and animals share basic neurobiological mechanisms associated with the complex behaviors that mimic mental disorders in animals (101).

Another difficulty posed to practitioners is that there are currently no international protocols guiding the administration of the diet; this is something that may be established from future research into KD. There was only one case study that detailed what the participant, diagnosed with schizophrenia, ate, and it was not established whether this individual was in ketosis. In the various studies in humans, outcomes were assessed following dietary durations that varied from 7 days to 2 years.

Further research into the neural correlates of KD is needed to help explain the mechanisms by which it acts. Some suggestions regarding methodologies, provided by Fusar-Poli are elaborated below. Changes in glucose metabolism seen in KD could be examined using positron emission tomography fluorodeoxyglucose. To observe the neural correlates of KD, a combination of electrophysiological measures including EEG and magnetoencephalogram and fMRI/PET to combine the high temporal resolution of the former with the high spatial resolution of the latter may be used (102).

In the neurological literature, a single study, in Alzheimer’s disease, used a synthesized ketogenic compound AC-1202 rather than a KD. AC-1202 is an MCT composed of glycerine and caprylic acid (23). It is not yet clear what role ketogenic pharmacotherapy options might play alongside or as a substitute for KD.

While these animal studies are placing research into KD on a firm footing and identifying some promising leads, on balance the evidence in humans is insufficient to form an opinion as to the efficacy or lack thereof of this intervention in the mental disorders reported. Further basic research to clarify the specifics of dietary manipulation or supplementation required to produce optimum ketosis in specific models is an obvious intermediate step toward studying the effectiveness of the diet in human mental disorders using conventional phases of research including open-label studies and randomized controlled trials.

## Author Contributions

EB derived the concept of the article from which she received supervision and expert advice in the area of psychiatry from KK and neurology from BT.

## Conflict of Interest Statement

The authors declare that the research was conducted in the absence of any commercial or financial relationships that could be construed as a potential conflict of interest.
